# A survey of canine filarial diseases of veterinary and public health significance in India

**DOI:** 10.1186/1756-3305-3-30

**Published:** 2010-04-08

**Authors:** Puteri Azaziah Megat Abd Rani, Peter J Irwin, Mukulesh Gatne, Glen T Coleman, Linda M McInnes, Rebecca J Traub

**Affiliations:** 1 School of Veterinary Science, The University of Queensland, Queensland 4072, Australia; 2 School of Veterinary and Biomedical Science, Murdoch University, Western Australia 6150, Australia; 3 Bombay Veterinary College, Maharastra Animal and Fisheries Sciences University, Parel, Mumbai 400012, India

## Abstract

**Background:**

*Dirofilaria *spp., *Acanthocheilonema *spp. and *Brugia *spp. have all been reported in Indian dogs. In previous studies, diagnosis was made by morphological identification only. This is the first geographically stratified cross-sectional study in India to determine the prevalence and geographical distribution of canine filarial species of veterinary and public health importance, using a combination of conventional and molecular diagnostic techniques.

**Results:**

A total of 139 from 525 dogs (26.5%; 95% CI 22.7, 30.3) were positive for microfilariae. The most common species of canine filaria identified in this study was *A. reconditum *(9.3%) followed by *D. repens *(6.7%) and *D. immitis *(1.5%). Three out of 525 dogs were found to have mixed infections on PCR. The morphological and molecular evidence on the sequence of the 18S gene and phylogenetic analysis of the ITS-2 region provided strong evidence that the canine microfilariae discovered in the Himalayan city of Ladakh belong to a novel species of *Acanthocheilonema*. Two dogs in Ladakh were also found to have mixed infections of the novel species described above and a unique microfilaria which morphologically resembled *Microfilaria auquieri *Foley, 1921.

**Conclusions:**

At least six species of filarial nematode are now known to infect dogs in India, two of which were reported for the first time in this study. The study also confirms and extends the geographical distribution of canine heartworm (*D. immitis*) which overlaps with *D. repens*, emphasising the importance for veterinary clinicians and diagnostic laboratories to utilise immunodiagnostic tests that will not cross-react between those two filarial species. From a public health viewpoint, the distribution and prevalences of these nematodes warrant an appropriate prophylaxis to be administered to dogs.

## Background

Filariasis in dogs is caused by several species of filariids. *Dirofilaria immitis*, the most pathogenic canine filarid is responsible for heartworm disease in dogs. Both *D. repens *and *Acanthocheilonema *spp. develop into adult worms in the subcutaneous tissue resulting in skin nodules. Adults of *Brugia *spp. are usually recovered from the mandibular, retropharyngeal and axillary lymphatics. Most infections *with D. repens*, *Acanthocheilonema *spp. and *Brugia *spp. are of minimal veterinary clinical significance, however all canine filariae have the potential to infect humans and remain important from a public health perspective [1,2].

Diagnostic methods for filarial infections include isolation of adult worms followed by morphological identification, morphological observation of circulating microfilariae by stained blood smears, direct wet smears, modified Knott's technique and the Wylie's filtration technique [2,3]. Histochemical or immuno-histochemical staining of circulating microfilariae has also been performed [4-6]. Detection of circulating antigen with commercial test kits is currently available and widely used for *D. immitis *[7,8]. Molecular diagnostic approaches are also increasingly utilised for research and surveillance purposes [9,10].

In India, *Dirofilaria *spp., *Acanthocheilonema *spp. and *Brugia *spp., have all been reported in dogs [6,11-13]. Previous reports on filarial infections in India are summarised in Table 1. Based on these limited number of studies, it is currently accepted that *D. immitis *is geographically restricted to India's north-east and *D. repens *to India's south, with an overlapping area centrally. There are no reports of *D. immitis *occurring elsewhere in India, despite anecdotal evidence to suggest its occurrence in Delhi (Sharma, personal communication, July 2008, Delhi). This accepted view is questionable as competent mosquito vectors for *D. immitis*, belonging to the genera *Culex, Aedes *and *Anopheles*, that also happen to act as vectors for *D. repens*, are present all over India [14]. Moreover, a case of human pulmonary dirofilariasis due to *D. immitis *was reported in Mumbai in 1989 [15], which casts further doubt on its currently accepted geographical distribution. Recently, 16 out of 75 microfilaraemic dogs were shown to harbour *B. malayi *by researchers at the Kerala Agricultural University's College of Veterinary and Animal Sciences using morphological and immunodiagnostic criteria [13]. However, since *B. ceylonensis *is endemic in Sri Lanka this finding needs to be confirmed using PCR as the microfilariae cannot be differentiated morphologically from *B. malayi *and it is likely the ELISA test cross-reacts among *Brugia *spp. [16].

**Table 1:** Previous reported prevalences of canine filarial species in different location in India

	Northeast India			Southern India	
	Mizoram n = 240	Orissa n = 7	Kolkata n = 3200	Kerala n = 160	Karnataka n = 400
*Dirofilaria immitis*	34%	57%	3%	0%	0%
*Dirofilaria repens*	0%	14%	0%	7%	21%

*Data obtained from [52,29,24,30,6]

In previous reported studies, microfilarial identification relied on morphological assessment only. Despite the availability of published measurements of various microfilariae, the inadequacy of morphological diagnosis was demonstrated by Rishniw and colleagues (2006) [9] when microfilariae initially identified as *A. reconditum *were later determined to be *D. immitis *by molecular methods. Morphological identification of microfilariae not only requires experienced personnel but it may be difficult to detect multiple infections with more than one species of filarial worms [2].

A geographically stratified cross-sectional study was undertaken to determine the prevalence and geographical distribution of canine filarial species of veterinary and public health importance in India using a combination of conventional and molecular diagnostic techniques.

## Methods

### Study site

India features a wide range of climatic zones, ranging from montane (cold, wet alpine regions) and semi-arid regions to the wet tropics. The ecology of vectors of medical and veterinary importance (and therefore the diseases they transmit) is highly dependent on climate [17]. The study was stratified to include four climatic zones of India. The locations of the places sampled represent a unique climatic condition of their own, based on information produced by The World Meteorological Organization [18]. Ladakh in India's far north (3000 m altitude), experiences a temperature that rarely exceeds 27°C in summer, while in winter temperatures drop to minus 20°C. Mumbai's climate can be described as tropical with a high level of humidity. The mean average temperature for Mumbai ranges from 16°C during winter to 30°C in summer. The climate of Delhi is a monsoon-influenced humid subtropical climate with average temperatures range from 7°C during its dry winter to 39°C in summer. Sikkim's climate can be described as subtropical highland with mild temperatures range from 25°C in summer to 4°C in winter [19].

**Figure 1:**
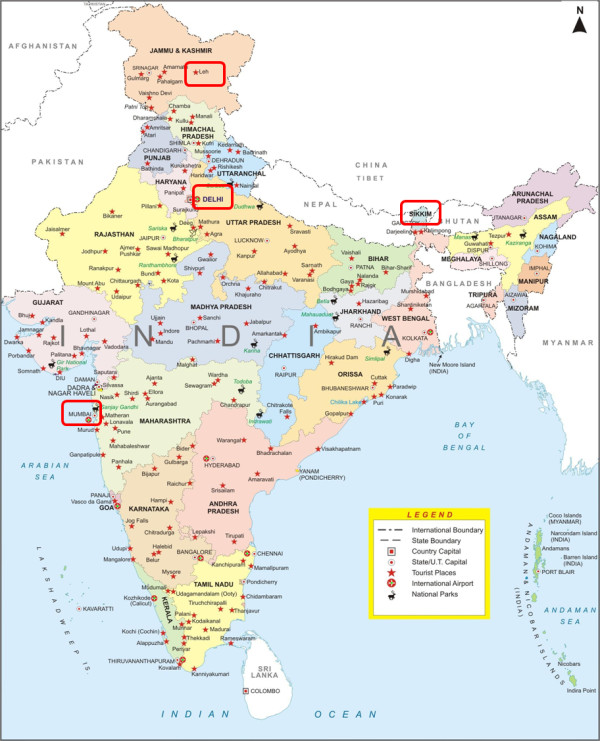
**Political map of India**. Areas outlined in red rectangles indicate sampling locations.

To facilitate the fieldwork, collaborations were established with Vets Beyond Borders, Jeevaashram, Krishnaashram, Bombay Veterinary College and In Defence of Animals India. These organisations allowed us access to stray and refuge dogs through their Animal Birth Control (ABC) and rabies vaccination programs. In these programs stray dogs are impounded, vaccinated, surgically neutered and released back to their original location [20]. The purpose of this program is to stabilise the street dog population and to help control the spread of rabies. Capillary and whole blood samples were collected from 525 dogs from four different cities (Figure 1), namely Gangtok and Jorethang in Sikkim (n = 101), Ladakh (n = 100) in Jammu and Kashmir, Delhi (n = 162) and Mumbai (n = 162). Blood samples were subjected to normal-thin and buffy-coat smears, air-dried and fixed in 100% ethanol and later stained with Giemsa for microscopic screening. Whole blood samples were also applied onto QIAcard FTA^® ^Four Spots (Qiagen) for molecular-based screening later.

### Microscopic examination of blood films

Stained blood films were examined under ×200 and ×400 objective lens for microfilariae. The microfilariae were measured using Olympus BH-2 microscope (Japan) calibrated eye micrometer and photographed using Olympus DP12 digital microscope camera (Japan).

### DNA extraction of adult D. immitis and blood from QIAcard FTA^®^

DNA from an adult worm of *D. immitis *(courtesy of Murdoch University) was extracted using the tissue sample protocol of the MasterPure DNA purification kit (Epicentre) according to the manufacturer's instructions and utilised as a positive control for this study.

Approximately 500 μl of blood from each animal was applied on QIAcard FTA^® ^Four Spots (Qiagen) which were cut into two cm strips (vertically) and allowed to air dry. A modified DNA purification protocol for tissue samples using the MasterPure DNA purification kit (Epicentre) was used to extract DNA from FTA cards. A 2 cm^2 ^piece of dried FTA card impregnated with a blood sample was cut into small pieces using a sterile scalpel blade on a clean microscope slide and placed in a 1.5 ml microcentrifuge tube. One μl of Proteinase K, 150 μl tissue lysis buffer and 150 μl phosphate buffer saline were added to the FTA card sample and incubated overnight at 65°C. The lysed sample was then cooled on ice for 5 minutes, 175 μl of MPC protein precipitation reagent added and the sample centrifuged at 6, 800 g for 10 minutes. Following centrifugation the supernatant was transferred into a clean 1.5 ml tube and 500 μl of isopropanol added. The sample was then inverted 30-40 times to promote DNA precipitation, centrifuged at 6,800 g for 10 minute and the supernatant removed carefully so as to not dislodge the DNA pellet. DNA pellet was washed twice with 70% ethanol. The sample was air dried for 5 minute to remove any remaining ethanol before the DNA pellet was resuspended in 20 μl of TE buffer.

### PCR assays and DNA sequencing

A single-step multiplex PCR targeted at amplifying the internal transcribed spacer-2 region of ribosomal DNA developed by Rishniw and colleagues (2006) [9] was utilised for molecular screening of canine filarial species in blood. Pan-filarial primers, forward: DIDR-F1 5'-AGT GCG AAT TGC AGA CGC ATT GAG-3' and reverse: DIDR-R1 5'-AGC GGG TAA TCA CGA CTG AGT TGA-3' were utilized to amplify and differentiate *D. immitis*, *D. repens*, *B. malayi*, *B. pahangi*, *A. reconditum *and *A. dracunculoides*. The PCR assay was carried out in a final volume of 25 μl containing 1 × PCR buffer, 1.5 mM MgCl_2_, 200 μM dNTPs, 0.5 μM of each primer, 1 U of Taq polymerase and 1 μl of DNA template. The PCR procedure was executed according to Rishniw et al. (2006). The PCR products were run on a 2% agarose gel in 1 × TAE buffer at 100V and visualised using Geldoc (Biorad). The anticipated product sizes of each species of microfilaria are listed in Table 2.

**Table 2:** Primer sequences used to amplify PCR products from filarial and canine blood samples [9].

Primer pair	Primer sequence	Gene target	Product origin	Product size (based-pairs)
DIDR-F1	AGT GCG AAT TGC AGA CGC ATT GAG	5.8S-ITS2-28S	*D. immitis*	542
DIDR-R1	AGC GGG TAA TCA CGA CTG AGT TGA		*D. repens*	484
			*B. malayi*	615
			*B. pahangi*	664
			*A. reconditum*	578
			*A. dracunculoides*	584

Product size and species of filarial nematodes amplified are also reported.

PCR products from 30% of all positive samples were purified using Qiagen spin columns (Qiagen). When a multiple-band product was obtained, target bands were excised and purified with Qiaquick Gel Extraction kit (Qiagen) prior to DNA sequencing. DNA sequencing was performed using an ABI 3130xl Genetic Analyzer (Applied Biosystems) with Big Dye 3.0 chemistry, after which sequences were edited and assembled using Finch TV (Geospiza Inc.).

### Primer design for 18S gene and amplification

A nested PCR was designed to amplify a partial region of the 18S rDNA of canine filarial species. Sequences of the near complete 18S rDNA of *B. malayi *[GenBank: AF036588], *Wuchereria bancrofti *[GenBank: AF227234] *D. immitis *[GenBank: AF036638] and *Dipetalonema *sp. [GenBank: DQ531723.1] were aligned using Clustal W http://align.genome.jp/ and an external and an internal set of primers (Table 3) were designed to amplify an approximately 800 bp and 700 bp product, respectively.

**Table 3:** External and internal primer sets for the amplification of a partial region of the 18S gene of most filarial species.

Primer name	Sequence (5'-3')
PAFilariaF1 (external)	GGTGAAACCGCGAACGGCTC
PAFilariaR1 (external)	CCGTCCCTCTTAACCATTATC
PAFilariaF2 (internal)	CTATAATGTACTTGATGTTGATTATC
PAFilariaR2 (internal)	CCATGCACGAGTATTCAGGCA

The PCR assay was carried out in a volume of 25 μl containing 1 × PCR buffer (Qiagen), 1.5 mM MgCl_2_, 200 μM of each dNTP, 0.5 μM of each of the forward and reverse primers, 1 U of Taq DNA polymerase (Qiagen) and 1 μl of extracted DNA. Five microlitres of Q-solution (Qiagen) was also added to optimise the PCR. The PCR conditions of both primary and secondary PCR are as follows: an initial activation step at 94°C for 2 min was followed by 35 cycles of amplification (94°C for 30 s, 56°C for 30 s and 72°C for 30 s) followed by a final extension step of 72°C for 7 min. The template for the secondary PCR amplification consisted of 1 μl of amplicon from the primary amplification.

### Phylogenetic analyses

Neighbour joining analyses were conducted with Tamura-Nei parameter distance estimates and trees constructed using Mega 4.1 software. Bootstrap analyses were conducted using 1000 replicates.

## Results

Out of the 525 dogs examined for circulating microfilaria, 27 (5.1%; 95% CI 3.3, 7.0) were positive using microscopic screening and 139 (26.5%; 95% CI 22.7, 30.3) confirmed for at least one filarial species by PCR. The most common species of canine filarial parasite identified in this study was *A. reconditum *followed by *D. repens *and *D. immitis*. The prevalence and geographical distribution of canine filarial species using both morphology and multiplex PCR are summarised in Table 4.

**Table 4:** The prevalences of canine filarial species in different cities in India by PCR and microscopy (in parentheses)

	Delhi n = 162	Mumbai n = 162	Sikkim n = 101	Ladakh n = 100	Overall prevalence n = 527
***Dirofilaria immitis***	4.3% (0%)	0% (0%)	1% (0%)	0% (0%)	**1.5% (0%)**
***Dirofilaria repens***	4.9% (0%)	16.7% (8%)	0% (0%)	0% (0%)	**6.6% (2.2%)**
***Acanthocheilonema reconditum***	22.2% (0.6%)	4.3% (1.2%)	5.9% (0%)	0% (0%)	**9.3% (0.6%)**
***Novel *spp.**	0% (0%)	0% (0%)	0% (0%)	48% (13%)	**9.1% (2.5%)**
***Microfilaria auquieri***	0% (0%)	0% (0%)	0% (0%)	0% (2%)	**0% (0.4%)**

### Morphology identification

**Figure 2:**
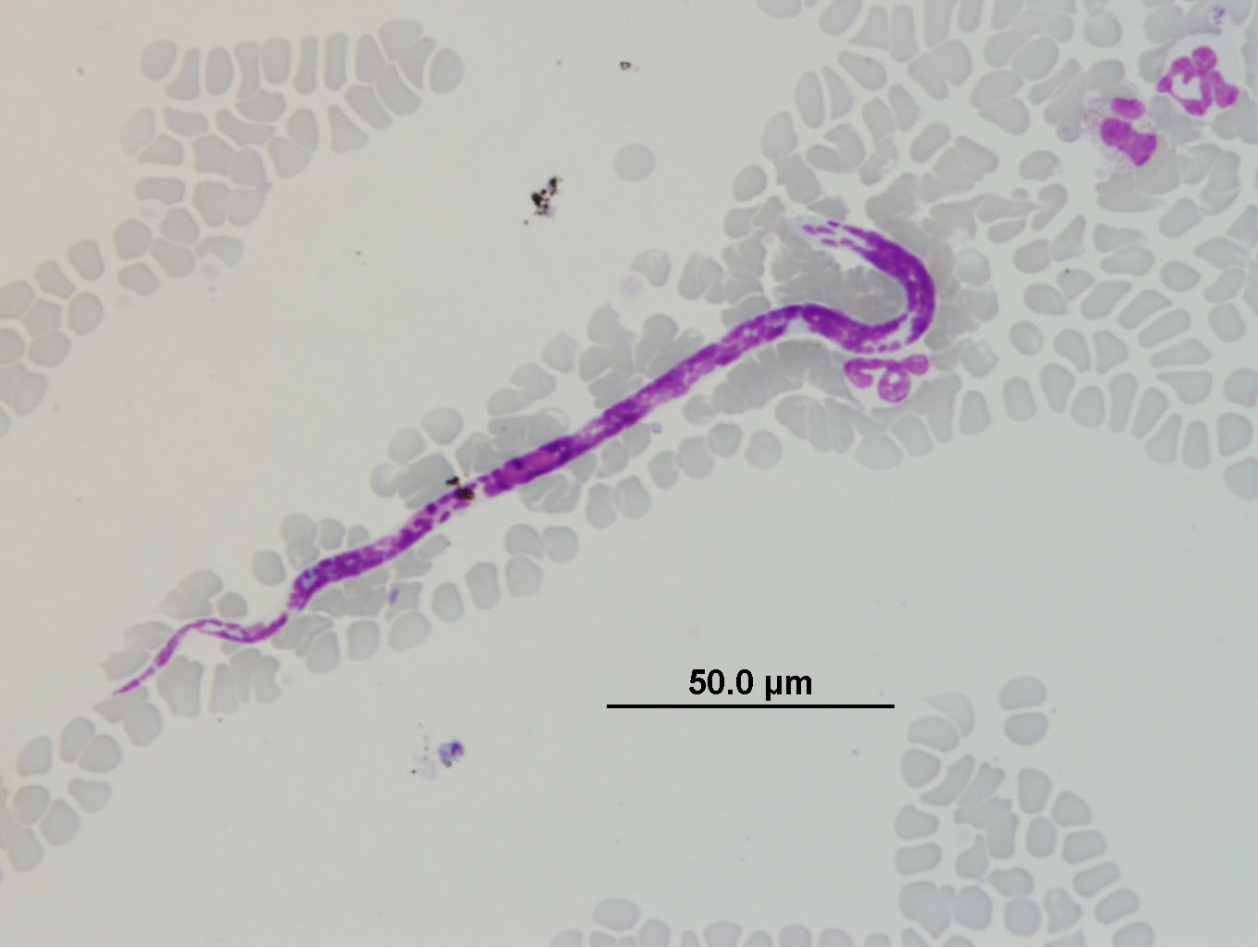
**Unidentified microfilaria observed in Giemsa blood smears of dogs from Ladakh**.

**Figure 3:**
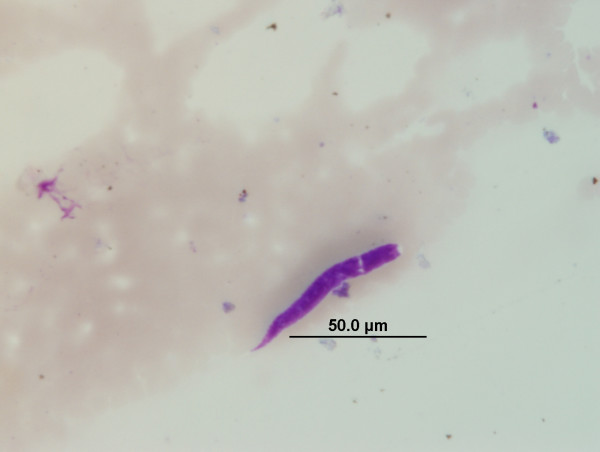
**Microfilaria observed in Giemsa blood smears of dogs from Ladakh, India which conform to the morphological descriptions of *Microfilaria auquieri *Foley, 1921**.

All microfilariae found in this study except for those identified from dogs in Ladakh could be identified based on previously described and published morphological criteria. In Ladakh, 13 dogs were found to harbour unsheathed microfilariae (Figure 2) with an average length of 165 μm (range 130 μm to 180 μm), that did not match the length of previously described canine microfilariae (Table 5). Two dogs in Ladakh were also found to have mixed infections of the unidentified microfilaria described above and another microfilaria of an unusual appearance (Figure 3) which was identified morphologically and presumed as *Microfilaria (Mf.) auquieri*, a species previously described by Foley [21] and Rioche [22] in Algeria, North Africa. Further analyses with regard to the genetic identity of the unidentified canine microfilariae in Ladakh were conducted using the 18S and ITS-2 genes.

**Table 5:** Measurements of microfilaria recorded by various author^a^

Filarial species	Special features of microfilaria	Microfilaria	
		Length (μm)	Width (μm)
*Dirofilaria immitis*	Unsheathed, tapered head, relatively straight tail	218 - 329	5.4 - 6.2
*Dirofilaria repens*		283 - 360	7.1 - 8.3
*Acanthocheilonema reconditum*	Unsheathed, round curved body, cephalic hook, blunt anterior end	250 - 270	4 - 4.5
*Acanthocheilonema dracunculoides*		195 - 230	Not available
*Cercopithifilaria grassi*		567	Not available
*Microfilaria auquieri*	Unsheathed	58 - 102	Not available
*Microfilaria ochmanni*	Sheathed	320	Not available
*Brugia malayi*	Sheathed, cephalic space: 6.3 - 6.7 μm	254 - 234	5.99-7.99
*Brugia pahangi*	Sheathed, cephalic space: 6.4 μm	200 - 189	4 - 5
*Brugia ceylonensis*	Sheathed, blunt tail, cephalic space: 6.3 - 6.7 μm	220 - 275	Not available

^a^Data obtained from [2,6,21,53,54]

### Single-step multiplex PCR

Out of 525 samples, 139 produced amplicons corresponding to *D. immitis, D. repens *or *A. reconditum*. Three out of 527 dogs were found to have mixed infections based on the amplicon sizes; one from the city of Mumbai with *D. repens *and *A. reconditum*, and two from the city of Delhi with *D. immitis *and *A. reconditum*, and *D. immitis *with *D. repens*. Two samples from Ladakh which found to have mixed infections by microscopic screening with presumably *Mf. auquieri*, only produced a single amplicon corresponds to *A. reconditum*. All PCR products amplified and sequenced at the ITS-2 region, except those from Ladakh as well as *A. reconditum *isolates from Delhi and Mumbai had their multiplex PCR results confirmed with published sequences for *D. immitis *from Mizoram, India [GenBank: EU087699], *D. repens *from Kerala, India [GenBank: FJ717410] and *A. reconditum *from Taiwan [GenBank: AF217801] with 99-100% homology on the basic local alignment search tool, BLASTn http://blast.ncbi.nlm.nih.gov/Blast.cgi.

*Acanthocheilonema reconditum *isolates from Delhi and Mumbai displayed 95% and 90% homology to the published ITS-2 sequence for *A. reconditum *from Taiwan [GenBank: AF217801]. Sequences for the ITS-2 region of *Acanthocheilonema *isolates from each city; Delhi, Mumbai and Ladakh were submitted to GenBank under accession numbers GU593976, GU593978 and GU593978, respectively.

### Genetic characterisation of unidentified canine microfilaria species

**Figure 4:**
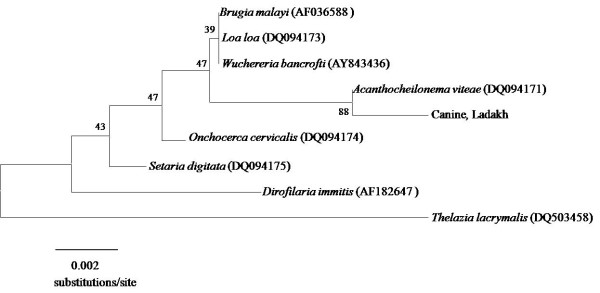
**Phylogenetic placement of the unidentified species of microfilaria from Ladakhi dogs based on partial SSU rDNA gene sequences**. Bootstrap values at nodes indicate percentagecalculated in 1000 replicates. *Thelazia lacrymalis *was used as an outgroup.

Clear and readable sequences spanning a 441 bp region of the SSU rDNA gene were obtained from a single Ladakhi isolate of the unidentified species of canine microfilaria. These were aligned and compared with previously published sequences of closely related filariid species *B. malayi *[GenBank: AF036588], *Loa loa *[GenBank DQ094173], *W. bancrofti *[GenBank: AY843436], *A. vitae *[GenBank: DQ094171], *Onchocerca cervicalis *[GenBank: DQ094174], *Setaria digitata *[GenBank: DQ094175] and *D. immitis *[GenBank: AF182647] using Clustal W (BioEdit v 7.0.5.3). *Thelazia lacrymalis *[GenBank: DQ503458] was used as an outgroup [23]. Although bootstrap support was low for the differentiation of all genera of filariid, the unidentified microfilariae isolated from dogs in Ladakh were distinctly placed within the same clade as *A. vitae *(Figure 4).

ITS-2 sequences of *Acanthocheilonema *were found to have identical sequence homology within the same geographical area. Sequences of six of the unidentified microfilaria isolates from Ladakh, two isolates of *A. reconditum *from Mumbai and Delhi and a single isolate from Sikkim, together with GenBank reference sequences of canine filarial species *A. reconditum *[GenBank: AF217801], *A. dracunculoides *[GenBank: DQ018785] were compared and aligned with a using Clustal W (BioEdit v 7.0.5.3).

**Figure 5:**
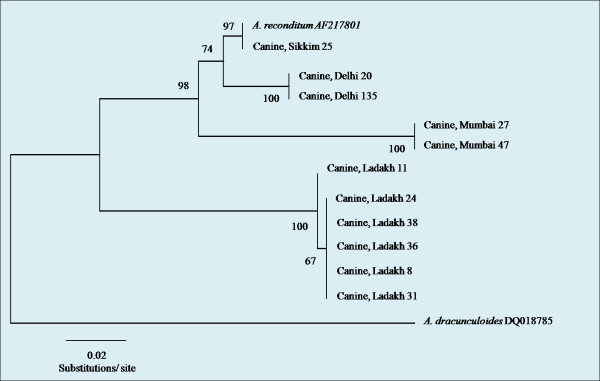
**An unrooted phenogram of the ITS-2 region of the unidentified species of microfilariae from Ladakh using neighbour-joining analysis with the Tamura-Nei model**. Bootstrap values at nodes indicatepercentage calculated in 1000 replicates.

The topology of the unrooted phenogram recognises five major groups within the genus *Acanthocheilonema*, which encompassed *A. reconditum *from Taiwan/Sikkim, *A. reconditum *from Delhi, *A. reconditum *from Mumbai, *Acanthocheilonema *isolates from Ladakh and *A. dracunculoides *as separate groups. Isolates from Mumbai formed a sister group to *A. reconditum *isolates from Taiwan, Sikkim and Delhi. There was very strong bootstrap placement for all six *Acanthocheilonema *isolates from Ladakh into a distinct group from all isolates of *A. reconditum *as well as *A. dracunculoides *(Figure 5).

Genetic distances of the ITS-2 region between *A. reconditum *and *Acanthocheilonema *isolates from Ladakh (11%) and Mumbai (9%) were similar to that between *A. reconditum *and *A. dranunculoides *(19%) (Table 6), whereas those between *A. reconditum *and isolates from Delhi were significantly less (3%).

**Table 6:** Distance matrix showing the nucleotide difference among ITS-2 gene sequences for microfilaria isolated from dogs from Delhi, Mumbai, Sikkim and Leh with reference sequences from GenBank for *A. reconditum *[GenBank: AF217801] and *A. dracunculoides *[GenBank: DQ018785].

*A. dracunculoides*_DQ018785					
*A. reconditum*_AF217801	0.19				
Canine, Delhi isolate (A.r)	0.20	0.03			
Canine, Ladakh isolate	0.22	0.11	0.13		
Canine, Sikkim isolate (A.r)	0.19	0.00	0.03	0.11	
Canine, Mumbai isolate (A.r)	0.26	0.09	0.09	0.17	0.09

Isolates from the study designated 'A.r' in parentheses are those that morphologically resembled *A. reconditum*.

## Discussion

This is the first comprehensive study that has utilised a combination of conventional and molecular techniques to determine the distribution and occurrence of canine filarial species in India. Despite anecdotal accounts of heartworm being present in Delhi, this study is the first to confirm its presence in that city, together with *D. repens*. This study is in agreement with previous reports [6,11,24] demonstrating a low prevalence of microfilaraemia overall, and that the most common filarial species found in India are *A. reconditum *and *D. repens*. Despite the relatively limited geographical locations used in this study, our results also support the aforementioned hypothesis that canine heartworm is primarily confined to tropical and sub-tropical areas of northern India, but seems to be absent towards the south. This hypothesis is debatable since competent mosquito vectors for *D. immitis *are present throughout central and southern India. For example, *Aedes albopictus *[25-27], a competent vector for *D. immitis *is present in Maharastra, Karnataka and Pondicherry [28], and heartworm is yet to be reported in dogs from these areas where climate would support larval development within the mosquito vector. It is noteworthy however that the prevalence of all filarial species determined in the current study may still an underestimate of its true value due to false negative test results. The high prevalence of *D. immitis *by necropsy that was reported in eastern and central India cannot be disregarded [29,30]. Authors of these studies also mentioned that more than 30% of dogs positive for *D. immitis *had occult infections, which is in agreement with studies from Australia in the 1980s [31]. Several types of occult filarial infection in dogs have been documented: pre-patent infection, naturally occurring unisexual infection and immune-mediated clearance of microfilariae, a similar situation that exists also in human filariasis [3,32-34]. PCR has been shown to detect occult infections of *Loa loa *in humans [35,36], however a recent study by Duscher (2009) has shown that a minimum parasitaemia level of 6 ± 0.43 microfilariae per 100 μl of blood on the FTA cards is need for detection of *D. repens *microfilariae by PCR detection [37]. The authors explain that it is hard to distribute large extracellular metazoan stages such as microfilariae on a filter paper, and this can therefore lead to false negative results for low parasitaemia infections. Furthermore, macrocyclic lactones such as ivermectin are known to possess microfilaricidal effects. Almost half the refuge dogs from Mumbai and Delhi sampled in this study had a history of receiving treatment with ivermectin for the control of gastrointestinal and ectoparasites. A recent study by Bazzocchi and colleagues [38] confirmed that with prolonged treatment of ivermectin, a significant decreased in circulating microfilariemia in dogs (less than 100 microfilariae/ml) occurred.

The most common filarial species found in dogs from this study are *A. reconditum *and *D. repens*. Most infections with *A. reconditum *and *D. repens *do not contribute to any clinical illness in dogs, but this is not the case in humans [1,2]. *Dirofilaria repens *causing subcutaneous nodules and sub-conjunctival infections in humans is now considered as a re-emerging zoonoses and it often leads to misdiagnosis of malignant tumours in endemic areas [1,24]. Although *D. repens *is endemic to a wide geographical area spanning Europe and Asia, human cases, often involving nodules in organs such as lungs, male genitals and female breast are most commonly reported from Italy and Sri Lanka [39]. With regard to *Acanthocheilonema*, only one human case has been reported in Australia, which involved the eye; the recovered worm morphological features were consistent with an unfertilised adult female *A. reconditum *[40]. *Dirofilaria immitis *infection in humans is rarely reported, but associated with pulmonary lesions or radiological coin lesions of the lung. The significance of *D. immitis *infection in human is the confusion and invariable radiological misdiagnosis of a primary or metastatic lung tumour, which usually leads to thoracotomy for open lung biopsy or wedge resection of the lung to obtain the correct diagnosis [41,42]. Sporadic reports of the immature heartworm in unusual locations such as the eye, mesentery, cerebral artery, spermatic cord and liver also exist [43-47]. To date, twelve cases of human subcutaneous dirofilariasis due to *D. repens *has been reported in southern India [24] and a single case of human pulmonary dirofilariasis due to *D. immitis *in Mumbai [15].

All microfilariae found in this study except for those identified from dogs in Ladakh could be identified based on previously described and published morphological criteria. Morphologic dimensions of the microfilariae genetically characterised as *A*. *reconditum *from Delhi, Mumbai and Sikkim matched previously documented measurements for *A. reconditum*. This was supported by molecular evidence to show that the isolates clustered within the *A. reconditum *group on phylogenetic analysis. Genetic distances of the ITS-2 region between *A. reconditum *and isolates from Delhi were within the range expected for intra-species variation of different geographical populations. However genetic distances of the ITS-2 region between *A. reconditum *and isolates from Mumbai were within the range expected for separate species of the same genera. Different species of filarial nematodes are reported to vary in their degree of intra-species variation based on the ITS-2 gene [48], making this comparative analysis difficult to confirm. Future molecular epidemiological studies comparing the intra-species variation of *Acanthocheilonema *spp. in different geographical locations at multiple loci is necessary to confirm this hypothesis. This finding is in contrast to the situation discovered with the microfilariae found to infect nearly half the dogs in Ladakh. Morphologically, the length of these latter microfilariae did not match any of the documented or known species of canine filariae. Molecular evidence based on the phylogenetic analysis of the 18S and ITS-2 regions provided strong evidence to show that the canine microfilariae found in Ladakh belong to a novel species of *Acanthocheilonema*. Genetic distances between this novel species and *A. reconditum *were within the range expected for separate species of the same genera. We propose that in the interim, this new species of microfilaria be designated *Acanthocheilonema ladakhii *until further and more detailed morphological, molecular and clinic-pathological studies are undertaken to describe and name this novel species of canine filarial parasite and ascertain its veterinary significance.

This study also presents the first report of what we presume to be *Mf. auquieri *in India and this serendipitous re-encounter with this filarial species after 50 years is of great parasitological interest. Although previously reported in stray dogs of Algeria, there is still much to be discovered about this enigmatic parasite. The adult form of *Mf. auquieri *is not yet known as attempts to search for the adult parasite failed despite a systematic post-mortem by the early French researchers [22]. It is interesting to note also that almost all dogs in Ladakh, and a small proportion in Delhi, were found infested with a species of blood-sucking fly (authors' personal observation), identified as *Hippobosca longipennis *syn. *capensis *Fabricius, 1805 [49], which is known to be an intermediate host and vector of *A. dracunculoides *[50]. Our findings in Ladakh have prompted us to speculate whether this same *Hippobosca *fly is also the vector/intermediate host for *Mf*. *auquieri *or whether it is just mere co-incidence that this fly also happens to be common in Algeria where *Mf. auquieri *was first reported [49].

As a side note the authors would like to add that the application of blood samples to filter paper-based technology, such as the FTA cards used in this study, allow for the rapid and safe dispatch of samples to diagnostic facilities capable of PCR-based diagnosis, with the added advantage of providing an archival potential. However, using this technique in humid regions can result in fungal contamination of the blood-impregnated filter paper as we have experienced during this study. A careful drying process of the papers is therefore recommended to prevent this problem from occurring.

## Conclusion

At least six species of filarial parasite are now known to infect dogs in India, two of which were reported for the first time in this study, namely *Mf. auquieri *and a novel species of *Acanthocheilonema*, both of which were discovered in the Himalayan city of Ladakh. The study also confirms and extends the known geographical distribution of canine heartworm in India. The distribution of heartworm in India extends from the Pakistani border in the west [51], Delhi and Sikkim in the north to the Burmese border in the east and Orissa to the south. The public health importance of *D. repens *also suggests that appropriate prophylaxis be administered to dogs throughout central and west India from as far south as Kerala to as far north as Delhi. From a diagnostic viewpoint, it is important to utilise an immunodiagnostic test that will not cross-react [8] between *D. immitis *and *D. repens *in areas where these two filarial species co-exist, for example, Delhi.

## Competing interests

The authors declare that they have no competing interests.

## Authors' contributions

PAMAR was involved in all phases of the study, including sampling and data collection, laboratory work, data analysis, intellectual interpretation, and writing the manuscript. RJT designed the study project, supervised the study, and was involved in sampling, field data collection, intellectual interpretation and critical revision of the manuscript for publication. LMM modified the DNA extraction method for FTA cards, construction and intellectual interpretation of the phylogenetic trees. PJI, MG and GTC supervised the study and were involved in intellectual interpretation and critical revision of the manuscript for publication. All authors read and approved the final manuscript.
